# Ovulation rates in a stair-step protocol with Letrozole vs clomiphene citrate in patients with polycystic ovarian syndrome

**DOI:** 10.1186/s40834-019-0102-4

**Published:** 2019-12-09

**Authors:** S. Thomas, I. Woo, J. Ho, T. Jones, R. Paulson, K. Chung, K. Bendikson

**Affiliations:** University of Southern California, 2020 Zonal Avenue, IRD 534, California, Los Angeles 90033 USA

## Abstract

**Purpose:**

To compare ovulation rates between Letrozole and Clomiphene Citrate (CC) using a stair-step protocol to achieve ovulation induction in women with Polycystic Ovarian Syndrome (PCOS).

**Methods:**

This is a retrospective cohort of predominantly Hispanic PCOS women of reproductive age who completed ovulation induction (OI) comparing women who underwent Letrozole stair-step protocol to those who underwent OI with CC stair-step. All women had a diagnosis of PCOS based on the 2003 Rotterdam criteria. For both protocols, sequentially higher doses of Letrozole or CC were given 7 days after the last dose if no dominant follicles were seen on ultrasonography. The primary outcome was ovulation rate (determined by presence of a dominant follicle) between the two treatment groups. Secondary outcomes included time to ovulation, clinical pregnancy rates and side effects.

**Results:**

49 PCOS patients completed a Letrozole stair-step cycle and 43 completed a CC stair-step cycle for OI. Overall, demographics were comparable between both groups. Ovulation rates with the Letrozole stair-step protocol were equivalent to CC stair-step protocol (96% vs 88%, *p* = 0.17). Although the mean time (days) to ovulation was shorter in the Letrozole group (19.5 vs 23.1, *p* = 0.027), the pregnancy rates were similar for both groups.

**Conclusions:**

This is the first study to date that has compared the efficacy of the stair-step protocol in PCOS patients using Letrozole and CC. Both Letrozole and CC can be prescribed in a stair-step fashion. Letrozole stair-step was as efficacious as CC stair-step; patients achieved comparable rates of ovulation and clinical pregnancy. Time to ovulation was shorter in the Letrozole protocol.

## Introduction

Polycystic ovary syndrome (PCOS) is one of the most common endocrine disorders in reproductive-aged women and the number one cause of infertility due to oligo-anovulation [1, 2]. Approximately 4 to 8% of reproductive age women have this metabolic disorder [3]. The first line fertility treatment for anovulatory women has been clomiphene citrate (CC) for ovulation induction for the past few decades. However, in a recent Cochrane review and a large randomized controlled trial (RCT), Letrozole, an aromatase inhibitor, was shown to lead to superior ovulation rates and live birth rates in women with PCOS when compared to CC [3, 4]. In the RCT, the ovulation rate for CC and Letrozole was 48.3% vs. 61.7% with a live birth rate of 19.1% vs. 27.5% respectively [4]. These studies have altered standard of practice, and now the first line treatment for anovulation in women with PCOS should include Letrozole.

As an aromatase inhibitor, Letrozole prevents the conversion of androgens to estrogen in the peripheral blood stream. The subsequent feedback to the hypothalamus containing reduced estrogen levels, triggers a compensatory increase in hypothalamic gonadotropin-releasing hormone (GnRH) secretion, and thus an increased release of pituitary gonadotropins (follicle stimulating hormone and luteinizing hormone). These gonadotropins subsequently promote growth of the follicles and stimulate ovulation. In contrast, CC is a selective estrogen receptor modulator (SERM). CC functions as an estrogen receptor antagonist in the hypothalamus, thus stimulating GnRH and subsequent FSH secretion.

The traditional protocol using Letrozole for ovulation induction is identical to the protocol used with CC [5]. Typical treatment begins with the lowest dose of the medication for 5 days starting on cycle day 3–5 after a spontaneous menses or after a progestin induced withdrawal bleed. If no ovulation is detected (by ultrasonography or mid luteal progesterone level) the patient undergoes a progestin withdrawal bleed to simulate normal menses and the dose is titrated systematically up with the next cycle and this continues until the maximum dose of medication is reached. The “stair step” protocol eliminates the use of progestin to induce a withdrawal bleed between sequential treatments. The time to ovulation is decreased because the progestin withdrawal step is eliminated, and an effective dose of the ovulation agent is found more quickly. Stair-Step protocols with CC for ovulation induction has been thoroughly explored [5–10]. These studies examined ovulation, and pregnancy rates using the stair-step protocols. Hurst and colleagues found the time to ovulation using CC was significantly shorter, by 32–53 days with the stair-step protocol compared with traditional regimen. In addition, they found higher dose-dependent ovulation rates [5]. This was likely due to the accumulation of medication in the body given the half-life of CC being approximately 5–7 days. There is limited published data on the time to ovulation and dose dependent rates of ovulation with Letrozole stair step.

We sought to examine the ovulation rates of Letrozole in a stair-step protocol compared to a similar stair-step protocol with CC in women with PCOS. We aimed to confirm whether improved ovulation rates with Letrozole are indeed higher than CC, which has been shown in previous studies using the standard protocol. We additionally aimed to assess incidence of side effects reported between the two treatment groups.

## Methods

We conducted a retrospective cohort study of women with PCOS who underwent Letrozole stair step for ovulation induction at a county hospital-based Reproductive Endocrinology and Infertility clinic at an academic institution. Our primary analysis included women ages 18–42 seen in clinic from January 2015 to January 2016 compared to a similar aged historic control of women with PCOS who underwent clomiphene citrate stair step from July 2013 to July 2014. The historical control group was a nested group with data previously collected from a large study at the institution. The participants were matched by diagnosis of PCOS, and treatment at the institution. The time frame was chosen as there was a institutional change in the treatment protocol for PCOS, with a transition from clomiphene citrate to Letrozole during this time frame. Per clinic standard policy, all patients undergoing ovulation induction were treated with a stair step protocol. Diagnosis of PCOS was based on the 2003 Rotterdam definition requiring oligo/anovulation and either the presence of clinical or biochemical signs of hyperandrogenism, oligoovulation/anovulation or polycystic ovaries, with exclusion of other causes of excess androgen activity. Women were excluded from the study if they underwent ovulation induction with medication other than Letrozole or CC during the indicated time periods. They were additionally excluded if they fell outside the pre-selected age ranges, had a Day 3 FSH > 10 or BMI > 40. Patients with BMI > 40 were excluded from any treatment with ovulation induction per clinic policy.

### Stair step protocol

**Fig. 1:**
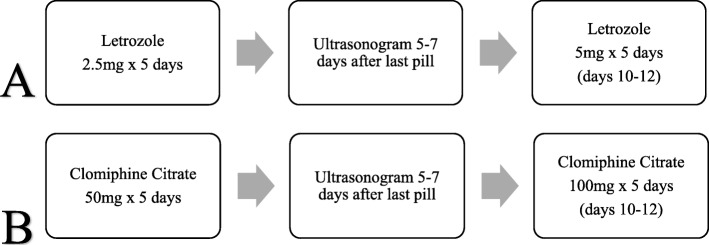
Flow diagrams for stair step protocols for (**a**) Letrozole and (**b**) Clomiphene Citrate for ovulation induction. Subsequent ultrasonogram was performed after “step up” in dosage until dominant follicle (18 mm) was seen or maximum dosage achieved per each treatment group

Women were prescribed the lowest dose of ovulation induction medication (50 mg CC or 2.5 mg Letrozole) for 5 days beginning with either menstrual cycle day 3–5 if they had spontaneous menses or were randomly started irrespective of past bleeding timing. Established doses of both medications were used in the respective stair-step protocols starting at the lowest dose: CC 50 mg increasing to 150 mg and up to 250 mg as needed; Letrozole 2.5 mg increasing to 5 mg and up to 7.5 mg as needed. A transvaginal ultrasound was performed approximately 1 week (5–7 days) after the last pill (Fig. 1.). If no response (all follicles < 10 mm) was noted on ultrasound, the patient was immediately given the sequential higher dose and an ultrasound was repeated in 1 week (5–7 days). The protocol was continued until a max of 7.5 mg for Letrozole or 250 mg for CC. Successful ovulation was defined with a positive ovulation predictor kit or ultrasound documentation of a preovulatory follicle of at least 18 mm that presumably would ovulate on its own. Documentation of ovulation type (spontaneous or triggered) was not performed in the CC group. Of note, when a 18 mm dominant follicle was noted on ultrasound, the patients were triggered with 10,000 IU HCG. Measurement of mid-luteal progesterone was not performed.

### Patient and cycle characteristics

Demographic information including age, gravity, parity, ethnicity, height, weight, BMI, protocol type, and hgbA1C was recorded for all the study participants. Endocrine dysfunction such as thyroid disorder was not examined in this study. However, it is clinic policy to rule out endocrine abnormalities in women with irregular menses, so if an abnormality in thyroid or prolactin for example were noted, treatment for that abnormality would have been initiated and adequately managed prior to ovulation induction. Side effects information was collected for both treatment regimens. However, given that the CC group was a historical cohort and has distinct anticipated side effects from the Letrozole group, the specific side effects assessed were different between the two groups. Intrauterine insemination (IUI) was added for patients with evidence of male factor by semen analysis results. Clinical pregnancy was defined as the presence of a fetal heartbeat at 6–7 weeks of pregnancy.

### Statistical analysis

Sample size was calculated using previously published data on rates of ovulation in both treatment arms, with ovulation rates of 88.5% for Letrozole and 76.6% for CC.^4^ With a power of 80% to detect a 10% difference in ovulation rates with a two-sided significant level of 0.05, we estimated we would need approximately 35 patients in each cohort. Differences between the two groups were analyzed by t-test for continuous variables, and chi-square or fisher’s exact test for categorical variables. Stata software, version 13 (Stata Corp, College Station, TX, USA) was used for all statistical analyses.

## Results

A total of 49 patient with PCOS completed the Letrozole stair-step protocol. The historical cohort whom underwent CC stair step protocol, included 43 patients. Overall, demographic variables were comparable between both groups (Table 1). The majority of women were Hispanic in both groups, although there was a larger proportion of Hispanic women in the Letrozole group than CC (98% vs 72%, *p* < 0.001). The mean age was 32 in both groups. Although women in the Letrozole group had a higher BMI than the CC group, this was not statistically significant (30.9 kg/m^2^ vs 29.5 kg/m^2^, *p* = 0.19). There was no significant difference in levels of insulin resistance between the two groups based on similar mean HbA1C levels (5.7% vs 5.6%, *p* = 0.17). A larger proportion of women in the CC group reported having experienced any side effects associated with treatment than the Letrozole group (41.9% vs 8.2%, *p* < 0.001). The side effects reported include bone/muscle pain, climacteric, headaches, gastrointestinal and fatigue. Clinical evidence of ovarian hyperstimulation syndrome (OHSS) was not documented for any of the patients in the study. In the Letrozole cohort, 31% (15/49) of patients received adjunctive therapy such as Metformin. Doses ranged from 1000 mg to 2000 mg daily. No patients were supplemented with steroids during treatment. Information on adjunctive therapy with Metformin for the CC group was not documented. Four patients in the Letrozole group had been treated with CC in the past. None of the CC patients had prior exposure to Letrozole.

**Table 1:** Patient Characteristics

Demographics	Letrozole	CC	*P*-value
# (%)	*n* = 49	*n* = 43	
Age	32.3 + 4.6	32.6 + 1.5	0.66
Hispanic	48 (98)	31 (72)	< 0.001
Nulligravida	24 (49)	26 (60.5)	0.27
Parous	9 (18.4)	7 (16.2)	0.79
BMI (kg/m^2^)^a^	30.9 + 4.7	29.5 + 1.5	0.19
Hgb A1C	5.6 + 0.4	5.7 + 0.7	0.17

^a^Data are mean + SD or n (%) unless otherwise specified. BMI: body mass index. Significance defined at *P* < 0.05

**Fig. 2:**
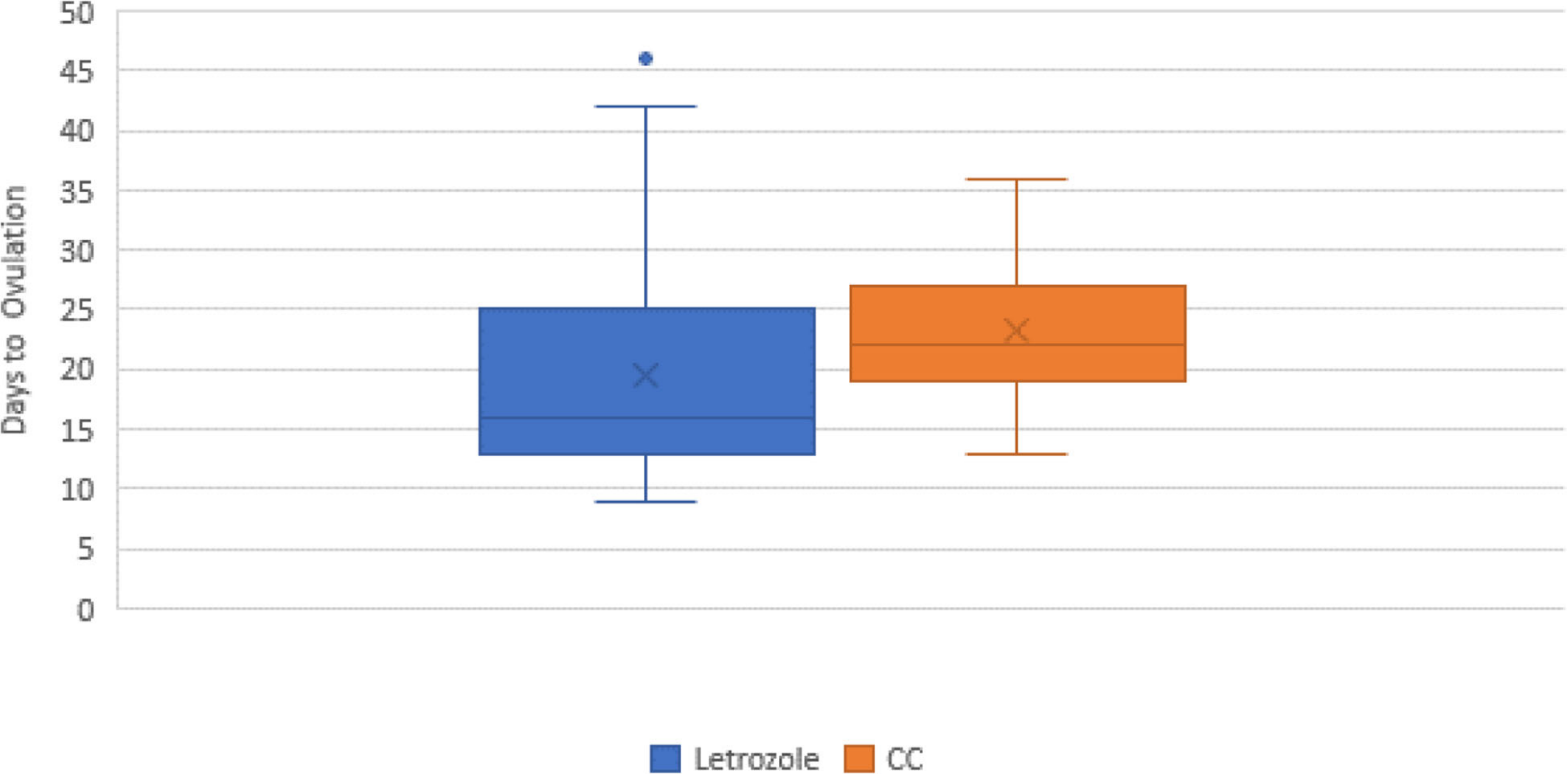
Comparison of mean time to ovulation between the CC and letrozole stair-step protocols. The letrozole shows overall lower mean time to ovulation (19.5 vs 23.1 days, *p* = 0.027)

The majority of patients ovulated under both Letrozole and CC stair step protocols, (96% vs 88%, *p* = 0.17), (Table 2). Data presented was only for the first treatment cycle per a patient. The mean time (days) to ovulation was shorter in the Letrozole group (19.5 vs 23.1, *p* = 0.027), (Fig. 2). When comparing ovulation rates by dose, there were no significant differences in ovulation rates at the lowest or highest doses between the two groups (data not shown). There was no difference in pregnancy rates in the Letrozole and CC groups, (6/49 [12.2%] vs 7/43 [16.3%], *p* = 0.58). There were no multiple gestations in both Letrozole and CC groups. Both the Letrozole and CC groups had a similar number of intrauterine insemination (IUI) procedures added to their cycles (16.3% vs 16.3%, *p* = 0.995).

**Table 2:** Ovulation Rates, Cycle Characteristics and Reproductive Outcomes

	Letrozole (n = 49)	CC (n = 43)	*P*-value
Overall ovulation	47 (96)	38 (88)	0.17
Mean time to ovulation (days)	19.5 + 9	23.1 + 9	0.027
Clinical pregnancies	6 (12.2)	7 (16.3)	0.58
IUI Added^a^	8 (16.3)	7 (16.3)	0.995
Number of Patients reporting Side Effects	4 (8.2)	18 (41.9)	< 0.001

^a.^ Data are n (%) unless otherwise specified. IUI: intrauterine insemination Significance defined at P < 0.05

## Discussion

The results of our study indicate that the Letrozole stair-step protocol yielded higher ovulation rates than the CC protocol for PCOS patients, although the results did not reach statistical significance (*p* = 0.17). We demonstrated that both Letrozole and CC can be prescribed in a stair-step fashion with equivalent cycle outcomes. Our study results revealed higher ovulation rates than previously published [5, 7]. These findings may be attributed to the unique patient population studied at a large urban center, and the primary outcome was measured by dominant follicle size leading to trigger of ovulation as opposed to confirmation of ovulation with an elevated serum progesterone. Additionally, the mean time for ovulation was shorter in the Letrozole group (19.5 days) than CC group (23.1 days). This reduced time to ovulation is consistent with prior studies examining the efficacy of a stair-step protocol compared to traditional ovulation induction protocols [5, 11]. Overall, Letrozole’s performance was comparable to CC in terms of ovulation rates and clinical pregnancy rates.

The stair-step protocol uses ultrasound to determine if the lowest dose is effective and requires immediate increase in dosage if there is an inadequate response, leading to a shorter time to ovulation and pregnancy, than in traditional protocols [5–7]. It has been proposed that the improved cycle outcomes in the stair-step protocol could be due to altered endometrial receptivity or additive effect of multiple doses of medication. It is important to note that all the stair-step or alternative protocols for ovulation induction have been studied with CC. There is limited data on ideal treatment regimens for Letrozole, especially in patients that don’t respond to initial low starting doses. There is concern that the cumulative effect of multiple doses of medication, could lead to a higher incidence of side effects. However, various studies on the stair-step protocol, have not shown increased rates of side effects compared to traditional regimens [5–7]. Results from our study revealed a higher rate in reported side effects in the CC group compared to the Letrozole.

Although there are adjuvant strategies to improve ovulation rates in PCOS patients such as diet/exercise, or supplemental medication including insulin sensitizing agents such as Metformin, these may prolong time to ovulation or pregnancy, as they require additional time to demonstrate biological effects on end organs [12–16]. There is evidence to support the use of laparoscopic ovarian drilling for improving ovulation rates in patients who have failed traditional ovulation inducing agents such as CC or Letrozole. However, surgery can lead to additional complications [17]. Treatment with gonadotropin medications can improve ovulation rates; yet gonadotropins are associated with significantly increased rates of higher order multiple pregnancy [18, 19]. Our study had no multiple gestations in both the Letrozole and CC stair-step protocols.

Letrozole has been demonstrated to offer improved performance compared to CC in PCOS patients, with significantly higher live birth and ovulation rates [4]. The reported mechanism is thought to be multi-factorial, including lower multi-follicular recruitment, and a lesser anti-estrogen effect on the endometrium. Results of these studies have altered standard practice in how to best achieve ovulation and subsequent pregnancy in patients with PCOS. However, Letrozole as an ovulation induction agent is still relatively new compared to CC. Thus, in comparison, there is less information on ideal cycle duration and dosage to achieve ovulation in anovulatory patients. Results from our study provide an alternative protocol for ovulation induction with Letrozole, while maintaining comparable cycle outcomes as CC.

Our study had several limitations. Although the Letrozole group was prospectively monitored, we utilized a historical control group, which were not completely matched for certain participant characteristics such as ethnicity. We excluded patients with BMI > 40 and those who did not have a diagnosis of PCOS. Thus, we are not able to generalize our results to all obese patients with chronic anovulation. Additionally, ovulation was triggered with HCG injection if a follicle measured at least 18 mm. We acknowledge that this does not accurately document ovulation, however for the purposes of this study, we presumed that if a dominant follicle forms that it would eventually ovulate. Although pregnancy would be the ideal end-point for determining ovulation, our study may not have been adequately powered to detect differences in secondary outcomes such as pregnancy rates. Adequately powered, prospective randomized trials are needed to further examine ovulation rates, cycle characteristics and pregnancy outcomes for Letrozole compared to CC in a stair-step protocol.

This is the first study to date that has compared the efficacy of the stair-step protocol in PCOS patients using Letrozole and CC. Our study revealed that the Letrozole stair-step protocol is as effective in inducing ovulation in PCOS patients as CC. Given the superiority of Letrozole to CC in inducing ovulation and higher live birth rates in PCOS patients, providers using Letrozole for ovulation induction in PCOS patients should consider utilizing the stair step protocol which is associated with shorter time to ovulation and minimal side effects.

## Data Availability

The datasets during and/or analyzed during the current study available from the corresponding author on reasonable request.
